# Diagnostic performance of two non-invasive biomarkers used individually and in sequential combination for cirrhosis associated with hepatitis C virus infection

**DOI:** 10.1038/s41598-022-24612-9

**Published:** 2022-11-23

**Authors:** Mohd Azri Mohd Suan, Huan Keat Chan, Xiaohui Sem, Sonjelle Shilton, Muhammad Radzi Abu Hassan

**Affiliations:** 1 Clinical Research Centre, Hospital Sultanah Bahiyah, Ministry of Health, Alor Setar, Kedah, Malaysia; 2 FIND, Geneva, Switzerland; 3 Office of Deputy Director General, Ministry of Health Malaysia, Putrajaya, Malaysia

**Keywords:** Biomarkers, Gastroenterology, Health care

## Abstract

This cross-sectional study evaluated the performance of the Aspartate Aminotransferase-to-Platelet Ratio Index (APRI) and the Fibrosis-4 (FIB-4) Index when they were used individually and in sequential combination to diagnose cirrhosis associated with hepatitis C virus infection. The final evaluation involved 906 people living with hepatitis C. The diagnostic performance of individual biomarkers at cut-off scores of 1.5 and 2.0 for the APRI and at 3.25 for the FIB-4 index was assessed. For the sequential combination method, the cirrhosis status of individuals with an APRI score between 1.0 and 1.5 were reassessed using the FIB-4. Transient elastography (TE) was used as the reference standard for diagnosing cirrhosis. The APRI, at a cut-off score of 1.5, showed a sensitivity, specificity, positive predictive value (PPV) and negative predictive value (NPV) of 44.9%, 97.6%, 91.1% and 76.3%, respectively. Increasing the cut-off score to 2.0 produced a much lower sensitivity (29.6%) and NPV (71.9%). The FIB-4, at a cut-off score of 3.25, yielded a sensitivity, specificity, PPV and NPV of 40.8%, 97.3%, 89.1% and 75.0%, respectively. The sequential combination method demonstrated a much more optimal diagnostic performance (50.2% sensitivity, 96.6% specificity, 89.0% PPV and 77.9% NPV). Overall, the APRI and FIB-4 Index performed better in diagnosing cirrhosis associated with hepatitis C when they were used in sequential combination.

## Introduction

The World Health Organization (WHO) set a goal to eliminate hepatitis C virus (HCV) as a public health threat by 2030^1^. HCV elimination requires a high degree of commitment and concerted efforts of all the countries to diagnose at least 90% of people living with the disease and treating at least 80% of them. Despite the availability of a national strategic plan for HCV in many countries, only 12 of them are on track to meet the HCV elimination goal^2^. While access to care remains a challenge to people living with HCV globally, the WHO recommends a simplified service delivery model incorporating elements of decentralisation, integration, and task-shifting^3^. Such a model is particularly suitable for low-and-middle-income countries, in which resources are generally limited and hepatitis C care is centralized at hospitals with gastroenterology specialty services^4,5^.

Following the advent of highly effective direct-acting antiviral (DAA) and rapid diagnostic kits, care decentralisation for HCV and task-shifting to non-specialists in primary healthcare (PHC) settings become feasible^6^. The findings from two recent systematic reviews suggest that decentralized treatment could produce a comparable cure rate in people living with the HCV^6,7^. However, it is often challenging to diagnose cirrhosis among potential treatment recipients in PHC settings due to the lack of facilities for imaging and biopsy^3,8^. Referring them to hospitals just for cirrhosis status assessment often results in loss to follow-up attributable to the stigma, logistical barriers and unaffordable transportation costs^9,10^.

Hence, the use of non-invasive biomarkers, such as the Aspartate Aminotransferase-to-platelet ratio index (APRI) and the Fibrosis-4 (FIB-4) index^11–13^, to assess the cirrhosis status of people living with HCV is becoming more common. APRI and FIB-4 scores are computed based on biochemical tests routinely performed in PHC settings and are thus deemed to be ideal alternatives to radiological measures for diagnosing cirrhosis. Some studies also proposed the combined use of the APRI and FIB-4 Index to enhance their accuracy^14,15^. As the demand for non-invasive and affordable tools for pre-treatment assessment in people living with hepatitis C is increasing, this study was conducted to evaluate the diagnostic performance of the APRI and the FIB-4 scores used individually and in sequential combination for diagnosing cirrhosis.

## Methods

### Study design and population

This cross-sectional study used the data originally collected for the Hepatitis C Elimination through Access to Diagnostics (HEAD-Start) study in Malaysia. The HEAD-Start study was a collaborative project between the Foundation for Innovative New Diagnostics (FIND), the Drugs for Neglected Diseases *initiative* (DND*i*) and the Ministry of Health (MOH), aiming to decentralize hepatitis C screening and treatment through 25 PHC clinics across Malaysia. It targeted adults aged between 18 and 70 years who sought primary care or harm reduction services and were found to have a positive serological rapid diagnostic test (RDT) result for HCV between December 2018 and December 2019^9^. Participants with a positive RDT result were then referred to one of the five selected tertiary hospitals for confirmatory diagnosis of hepatitis C (based on HCV ribonucleic acid (RNA) test using the reverse transcription polymerase chain reaction technique), laboratory biochemical tests and liver stiffness assessment using transient elastography (TE). Subsequently, those with a confirmed diagnosis of hepatitis C were offered DAAs-based treatment. Information on participant’s socio-demographic, taking any alcohol drinks, HIV, and hepatitis B infection status (HBV), laboratory test results and radiological findings were collected using a standardized data collection form. In the current study, only participants with a confirmed diagnosis of HCV and valid liver stiffness measurement (LSM) were included for the analysis. Those reporting a history of taking any alcohol drinks were excluded from the study.

### Laboratory analysis and calculation of APRI and FIB-4 scores

Based on the aspartate aminotransferase (AST) level, alanine aminotransferase (ALT) level and platelet count, APRI and FIB-4 scores for each individual were calculated using the following formulae^16,17^:

$${\text{APRI}}\;{\text{score}} = \frac{{(AST\;\left( {IU/L} \right)/ULN\;of\;AST\;(IU/L))}}{{Platelet\;count(10^{9} /L)}} \times 100$$

$$\mathrm{FIB}{\text{-}}4\,\mathrm{ score }= \frac{Age \left(years\right) \times AST (IU/L)}{Platelet\, count \left({10}^{9}/L\right) \times \sqrt{ALT}\left(IU/L\right)}$$

The upper limit of the normal AST level ranged between 34 and 50 IU/L, depending on the reference populations in each participating site.

**Figure 1:**
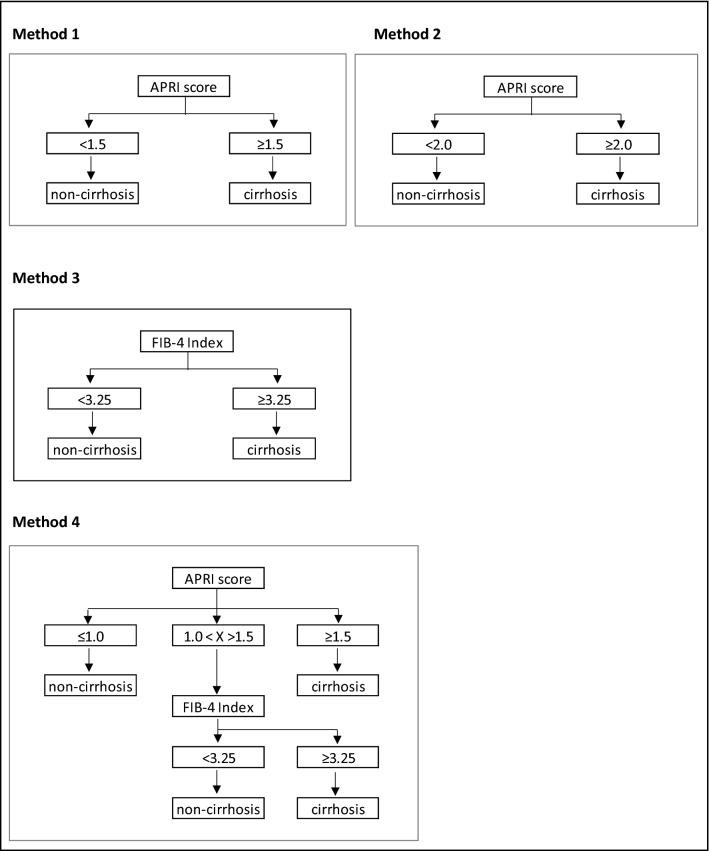
Methods of non-invasive biomarkers to diagnose liver cirrhosis using transient elastography as a reference standard. Method 1—APRI using a score of 1.5 as the binary cut-off. Method 2—APRI using 2.0 as the binary cut-off. Method 3—FIB-4 Index using 3.25 as the binary cut-off. Method 4 (the sequential combination method) –The cirrhosis status of those with an APRI score between 1.0 and 1.5 were reassessed using FIB-4 score. *APRI* aspartate-to-platelet ratio index, *FIB-4* fibrosis 4 index.

The commonly used cut-offs for APRI (≥ 1.5 and ≥ 2.0) and FIB-4 (≥ 3.25) scores were used to suggest the presence of cirrhosis^3^. This study also evaluated the sequential combination approach, under which the cirrhosis status of individuals with an APRI score between > 1.0 and < 1.5 were reassessed using a FIB-4 score (using 3.25 as the cut off score to suggest cirrhosis) (Fig. 1).

### Transient elastography

TE (FibroScan^©^), a non-invasive, ultrasound-based technology to measure the liver tissue stiffness, was used as the reference standard to diagnose cirrhosis in the current study^8^. The procedure was performed prior to DAAs treatment initiation by a trained physician or operator at each participating sites. The LSM, expressed in kilopascals (kPa), was based on a median value of at least ten successful measurements with an interquartile range of ≤ 30% and success rate of ≥ 60%. The diagnosis of cirrhosis was confirmed if the LSM was ≥ 12.5 kPa with the M probe or > 10.0 kPa with the XL probe^18,19^.

### Statistical analysis

The statistical analysis was performed using the R statistical software version 3.5.2^20^. Continuous variables were expressed as means and standard deviations if the data were normally distributed and analysed using the independent t-test. Variables with skewed data distribution were presented as median and interquartile range and analysed using the Mann–Whitney test. Categorical data was summarized as numbers and percentages and analysed using the Pearson’s chi-square or Fisher’s exact test. Receiver operating curves (ROC) were used to evaluate the ability of APRI and FIB-4 scores to discriminate between cirrhotic and non-cirrhotic participants. An area under ROC (AUROC) of 1.0 suggested a perfect discriminatory ability, ≥ 0.90 as excellent, ≥ 0.80 as good, ≥ 0.70 as fair, and < 0.70 as poor^21^. The aspects of diagnostic performance evaluated for the two biomarkers were their specificity, sensitivity, positive predictive value (PPV) and negative predictive value (NPV) when they were used individually and in sequential combination.

### Ethics approval and consent to participate

This study received approval from the Medical Research and Ethics Committee, Ministry of Health Malaysia (NMRR-18-2282-43132) and was conducted in accordance with the ethical standards of the 1964 Helsinki Declaration. All participants gave written informed consent prior to any study procedures.

## Results

### Baseline patient characteristics

Of the 1052 participants with a confirmed diagnosis of hepatitis C, two were excluded for missing data, 17 of no LSM and 127 for a history of taking alcohol drinks. The demographic and laboratory characteristics of the 906 individuals included in the current study are presented in Table 1. They were mainly male (93.4%) and had a mean age of 46.3 ± 9.63 years. Only 144 of them (15.9%) had HIV co-infection, and 1.2% had HBV infection. The median ALT level, AST level, platelet count and LSM were 56.0 ± 54.00 IU/L, 53.0 ± 41.00 IU/L, 221.5 ± 81.28 × 10^9^/L and 9.4 ± 9.60 kPa, respectively. The median APRI and FIB-4 scores were 0.6 ± 0.74 and 1.5 ± 1.49, respectively (Table 1).

**Table 1:** Baseline characteristics of the 906 individuals evaluated. *SD* Standard deviation, *APRI* aspartate aminotransferase to platelet ratio index, *FIB4* fibrosis 4 index, *ALT* alanine transaminase, *AST* aspartate aminotransferase, *HIV* human immunodeficiency virus, *HBV* hepatitis B virus, *a* Pearson chi-square test, *b* independent *t* test, *c* Mann–Whitney test.

Characteristics	Total	With cirrhosis	Without cirrhosis	*p*-value
**Gender, n (%)**				
Male	846 (93.4)	289 (90.0)	557 (95.2)	0.003^a^
Female	60 (6.6)	32 (10.0)	28 (4.8)	
Age, mean (SD)	46.3 (9.63)	48.8 (9.26)	44.9 (9.55)	< 0.001^b^
**HIV infection status, n (%)**				
Positive	144 (15.9)	31 (9.7)	113 (19.3)	< 0.001^a^
Negative	650 (71.7)	231 (72.0)	419 (71.6)	
Unsure/Never tested before	112 (12.4)	59 (18.4)	53 (9.1)	
**Hepatitis B infection status, n (%)**				
Positive	11 (1.2)	5 (1.6)	6 (1.0)	0.764^a^
Negative	259 (28.6)	90 (28.0)	169 (28.9)	
Unsure/Never tested before	636 (70.2)	226 (70.4)	410 (70.1)	
ALT (IU/L), median (IQR)	56.0 (54.00)	72.0 (66.00)	48.0 (42.00)	< 0.001^c^
AST (IU/L), median (IQR)	53.0 (41.00)	79.0 (63.00)	44.0 (27.00)	< 0.001^c^
Platelet (× 10^9^/L), mean (SD)	221.5 (81.28)	170.2 (71.45)	249.6 (72.10)	< 0.001^b^
Transient elastography (kPa), median (IQR)	9.4 (9.60)	20.9 (13.10)	7.3 (3.20)	< 0.001^c^
APRI Score, median (IQR)	0.6 (0.74)	1.3 (1.49)	0.5 (0.40)	< 0.001^c^
FIB-4 Score, median (IQR)	1.5 (1.49)	2.8 (2.65)	1.2 (0.86)	< 0.001^c^
**Liver fibrosis stage according to TE, n (%)**				
F0-1 (< 7.0 kPa)	264 (29.1)	–	–	–
F2 (7.0 – 9.4 kPa)	194 (21.4)	–	–	
F3 (9.5 – 12.4 kPa)	127 (14.0)	–	–	
F4 (≥ 12.5 kPa)	321 (35.4)	–	–	

Cirrhosis was more common in the male participants than in the female participants (90.0% versus 10.0%, *p* = 0.003). The participants with liver cirrhosis were significantly older. They also showed higher ALT level, AST level, LSM, APRI score and FIB-4 score but a lower platelet level.

### Liver cirrhosis status

A total of 321 (35.4%) participants were confirmed to have cirrhosis with a mean LSM of 24.2 kPa, while the rest had no cirrhosis with a mean LSM of 7.6 kPa (Table 2). When the APRI cut-off score was set at 1.5, 158 (17.4%) of them were suggested to have cirrhosis. A much lower number of individuals (102, 11.3%) were suggested to have cirrhosis if the APRI cut-off score was increased to 2.0. FIB-4 scores, at a cut-off of 3.25, suggested that 147 (16.2%) of the individuals had cirrhosis. The sequential combination method resulted in a higher percentage of individuals with cirrhosis (181, 20.0%). When using this method, the mean LSM for individuals with cirrhosis and non-cirrhosis were 26.0 kPa and 10.3 kPa, respectively.

**Table 2:** Distribution of cirrhotic status by transient elastography, APRI, FIB-4 and the sequential combination method (N = 906). *LSM* liver stiffness measurement, *TE* transient elastography, *AST* aspartate aminotransferase, *ALT* alanine transaminase, *SD* standard deviation, *APRI* aspartate aminotransferase to platelet ratio index, *FIB-4* fibrosis 4 index.

Characteristic	Sample count, n (%)	LSM (kPa), mean (SD)	AST (IU/L), mean (SD)	ALT (IU/L), mean (SD)	Platelet (× 10^9), mean (SD)	Age (years), mean (SD)
**LSM by TE**						
Cirrhosis (≥ 12.5 kPa)	321 (35.4)	24.2 (11.44)	93.5 (53.43)	93.1 (72.09)	170.2 (71.45)	48.8 (9.26)
Non-cirrhosis (< 12.5 kPa)	585 (64.6)	7.6 (2.22)	51.3 (32.69)	60.7 (54.63)	249.6 (72.10)	44.9 (9.55)
**APRI score I**						
Cirrhosis (≥ 1.5)	158 (17.4)	26.6 (13.08)	129.4 (65.65)	126.8 (101.13)	127.9 (58.51)	48.8 (8.96)
Non-cirrhosis (< 1.5)	748 (82.6)	10.7 (7.48)	52.9 (25.03)	60.7 (44.03)	241.3 (70.99)	45.7 (9.69)
**APRI score II**						
Cirrhosis (≥ 2.0)	102 (11.3)	29.3 (13.82)	144.6 (71.08)	131.5 (94.61)	112.2 (49.77)	49.1 (8.48)
Non-cirrhosis (< 2.0)	804 (88.7)	11.4 (8.18)	56.3 (29.33)	64.7 (53.7)	235.4 (73.64)	45.9 (9.71)
**FIB-4 Index**						
Cirrhosis (≥ 3.25)	147 (16.2)	26.7 (13.82)	116.1 (68.51)	96.4 (80.80)	114.9 (45.58)	52.2 (8.58)
Non-cirrhosis (< 3.25)	759 (83.8)	10.9 (7.55	56.6 (32.21)	67.5 (58.21)	242.1 (69.68)	45.1 (9.40)
**Sequential combination method**						
Cirrhosis	181 (20.0)	26.0 (13.39)	122.0 (64.62)	117.3 (97.88)	129.4 (56.00)	50.0 (9.36)
Non-cirrhosis	725 (80.0)	10.3 (6.88)	52.3 (24.97)	60.9 (44.59)	244.5 (69.49)	45.3 (9.47)

### Diagnostic performance of biomarkers

**Figure 2:**
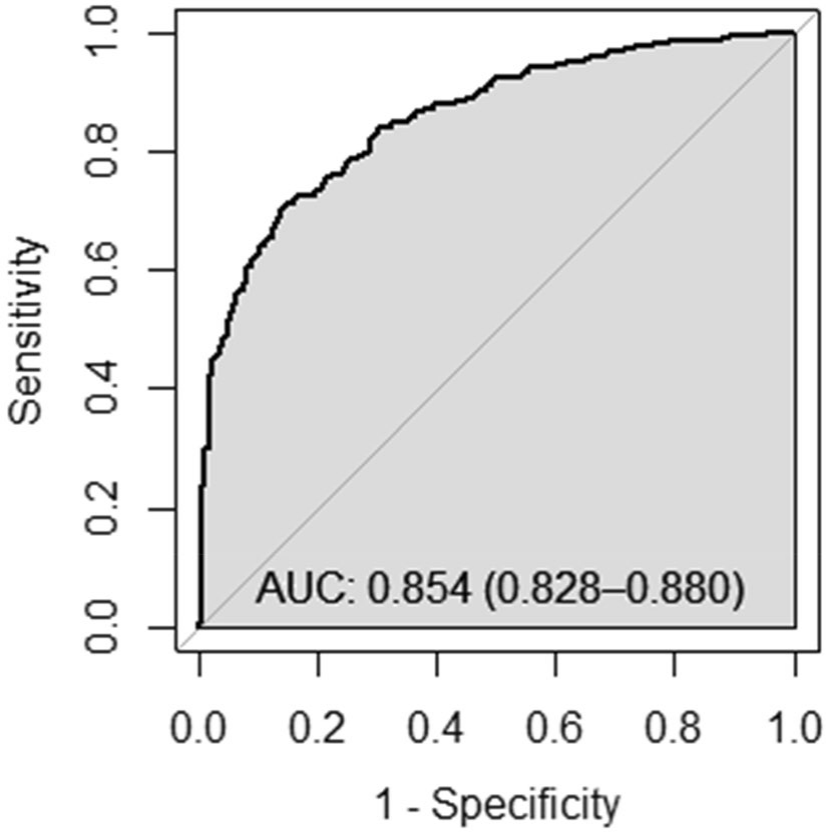
Receiver operating characteristic analysis for the accuracy of APRI to diagnose cirrhosis (transient elastography ≥ 12.5 kPa) in patients with hepatitis C infection (n = 906).

**Figure 3:**
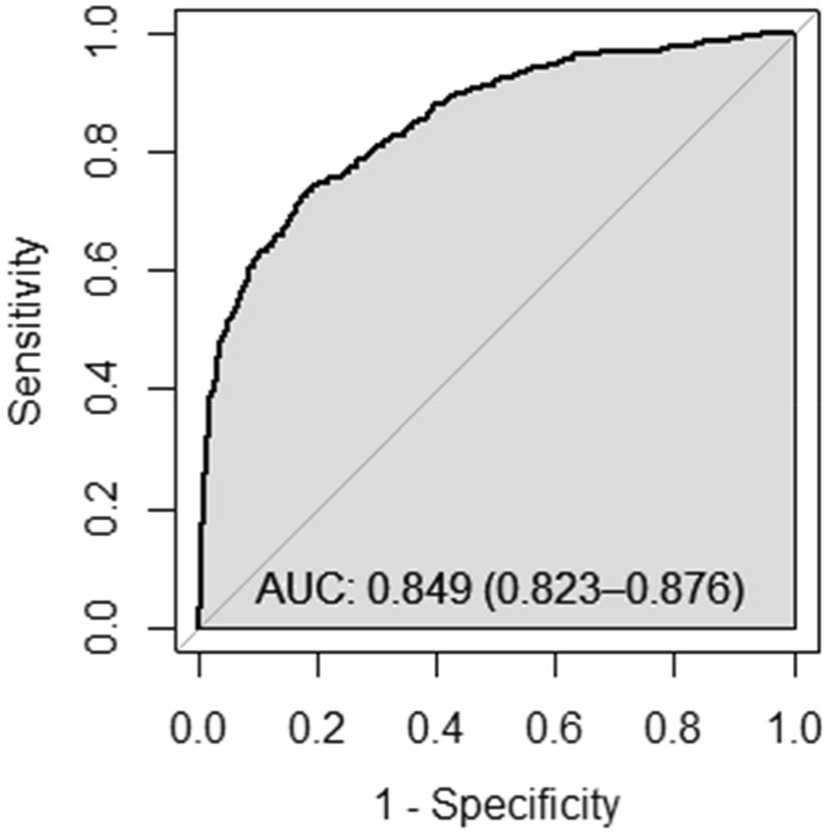
Receiver operating characteristic analysis for the accuracy of FIB-4 to diagnose cirrhosis (transient elastography ≥ 12.5 kPa) in patients with hepatitis C infection (n = 906).

Both the APRI and FIB-4 Index were generally able to discriminate between cirrhotic and non-cirrhotic cases, indicated by an AUROC of 0.854 and 0.849 (Figs. 2 and 3). The APRI with a cut-off score ≥ 1.5 produced a sensitivity of 44.9%, a specificity of 97.6%, a PPV of 91.1% and an NPV of 76.3%. Despite a better specificity (98.8%), the APRI with a cut-off score ≥2.0 showed the lowest sensitivity (29.6%) and NPV (71.9%) among all the methods. The FIB-4 Index with a cut-off score ≥ 3.25 yielded a sensitivity of 40.8%, a specificity of 97.3%, a PPV of 89.1% and an NPV of 75.0%. The sequential combination method showed a relatively optimal diagnostic performance with the lowest number of false negative cases. This method yielded a sensitivity, specificity, PPV and NPV of 50.2%, 96.6%, 89.0% and 77.9%, respectively (Table 3). Overall, the cirrhosis status of approximately 80.1% (726/906) of the individuals included in the current study matched the findings of TE when using the sequential combination method.

**Table 3:** Diagnostic performances of APRI, FIB-4 and the sequential combination method for liver cirrhosis among people living with hepatitis C. *TE* transient elastography, *PPV* positive predictive value, *NPV* negative predictive value, *APRI* aspartate-to-platelet ratio index, *FIB-4* fibrosis 4 index. *For patients with APRI score between > 1.0 and < 1.5, liver cirrhosis was predicted by further assessment using FIB-4 index.

Methods	Sensitivity (%)	Specificity (%)	PPV (%)	NPV (%)	No. of participants matched the finding of TE, n (%)	False positive cases, n (%)	False negative cases, n (%)
APRI with cut-off ≥ 1.5	44.9	97.6	91.1	76.3	715 (78.9)	14 (1.5)	177 (19.5)
APRI with cut-off ≥2.0	29.6	98.8	93.1	71.9	673 (74.3)	7 (0.8)	226 (24.9)
FIB-4 with cut-off ≥ 3.25	40.8	97.3	89.1	75.0	700 (77.3)	16 (1.8)	190 (21.0)
Sequential combination method*	50.2	96.6	89.0	77.9	726 (80.1)	20 (2.2)	160 (17.7)

## Discussion

To support the decentralisation of hepatitis C treatment, it is imperative to identify simple yet reliable tools for the diagnosis of liver cirrhosis that can be used at PHC level. A diagnostic strategy with good specificity, characterized by less false positive cases (serum biomarker calculation predicts cirrhosis but TE values less than 12.5), indicate the tools performed well in confirming liver cirrhosis. Conversely, if the tools resulted in many false negative cases (serum biomarker calculation predicts non-cirrhosis but TE values equal or more than 12.5), it could imply that the cases of probable liver cirrhosis would be missed and treatment failure is more likely to occur due to inappropriate treatment duration given. Within this context, underdiagnosis of liver cirrhosis and missed the cases (false-negative results) is more concerning than overdiagnosis (false-positive results).

This study showed that sequential combination method performed better when using TE as a reference standard, diagnosing 726 cases of liver cirrhosis, whereas using APRI or FIB-4 index alone captured a smaller number of cases with accurate diagnosis. Although all four methods used in this study had false-negative results for liver cirrhosis, but the rate was lower with sequential combination method (17.7%, 160 cases) than using APRI (19.5% with cut-off ≥ 1.5, 24.9% with cut-off ≥ 2.0) and FIB-4 (21.0%) alone.

The current study demonstrated good discriminatory ability for both the APRI and FIB-4 in diagnosing liver cirrhosis among people living with hepatitis C, evidenced by an AUROC above 0.8. The finding was consistent with those of most previous studies, supporting the ability of both tools to discriminate between cirrhosis and non-cirrhosis^14,17,22–24^. To the best knowledge of the authors, this is the first study evaluating four different tools in cirrhosis diagnosis including a sequential combination method. It was found that APRI with a cut-off score ≥2.0 and FIB-4 with a cut-off score ≥ 3.25 had lower sensitivity and NPV in diagnosing cirrhosis compared to APRI with a cut-off score ≥ 1.5. However, when applying the sequential combination method, a significant decrease in the number of false negative cases was observed in parallel with the elevated sensitivity and NPV. Such finding suggests that it is important to reassess the borderline cases with an APRI score between 1.0 and 1.5 to improve the accuracy of liver cirrhosis diagnosis among people living with HCV. It was shown in the current study that the FIB-4 can fill the gap and complement the APRI to enhance the diagnosis accuracy in such cases.

Variations in the APRI and FIB-4 scores result from the different parameters included in the formulae. As commonly used indicators for hepatocellular injury and impaired liver function, AST and ALT levels tend to rise in those who have liver cirrhosis^25,26^. A low platelet level is also often observed in people with liver cirrhosis, mainly as a result of platelet sequestration in the spleen and reduced production of thrombopoietin in the liver^27^. These would explain why the APRI did not perform as well as when it is used together with FIB-4, as the latter method includes more parameters in the calculation.

TE has been extensively studied for the assessment of cirrhosis among individuals with HCV infection. A meta-analysis showed that TE has performed well in differentiating cirrhosis and non-cirrhosis with an area under receiver operating characteristic of 94%^28^. Importantly, the operators need to comply with technical specifications (at least 10 successful measurements, success rate ≥ 60% and interquartile range of ≤ 30%) when performing LSM using TE to get a valid measurement. In the current study, physicians or operators performing the procedure were previously trained, and with strict criteria for reliable LSM as described in the methodology above, it will minimise inter-operator variability. Using TE as reference standard for diagnosing cirrhosis, APRI or FIB-4 alone only managed to accurately detect cirrhosis in less than 80% of total cases. This low diagnostic capacity of APRI and FIB-4 could be influenced by participants with HIV co-infection by factors such as antiretroviral drug-related hepatotoxicity and HIV-induced thrombocytopenia^29,30^.

The strengths of the current study include the large number of study subjects with a confirmed diagnosis of hepatitis C, as well as the high proportion of study subjects with liver cirrhosis. Instead of suggesting new cut-off scores for the APRI and the FIB-4 index to diagnose liver cirrhosis as in other studies^15^ and which are rarely adopted in the actual practice, the current study focused on evaluating the commonly used, pre-validated cut-off scores for both the biomarkers. However, this study was limited by the use of TE instead of liver biopsy for the diagnosis of cirrhosis. Liver biopsy remains the gold standard in diagnosing liver cirrhosis, but TE is an acceptable alternative due to its practicality, safety and non-invasive nature. The use of TE for liver staging is also in line with recommendation from the WHO^3^.

In conclusion, the sequential combination method demonstrated relatively good specificity with fewer false negative cases compared to when the APRI and the FIB-4 index were used individually in diagnosing liver cirrhosis among people living with hepatitis C. Therefore, this method can assist in diagnosing cirrhosis among potential treatment recipients at PHC settings where facilities for imaging and biopsy are lacking, and thus, facilitate the decentralisation of hepatitis C treatment.

## Data Availability

The datasets generated and analyzed during the current study are available from the corresponding author on reasonable request.

## References

[1] The World Health Organization. *Combating Hepatitis B and C to Reach Elimination by 2030: Advocacy Brief*. https://apps.who.int/iris/handle/10665/206453 (2016) (Accessed 23 May 2022).

[2] The CDA Foundation. *Just 12 Countries Worldwide on Track to Eliminate Hepatitis C Infection by 2030, with United Kingdom, Italy and Spain Among Those Joining The List.* https://cdafound.org/just-12-countries-worldwide-on-track-to-eliminate-hepatitis-c-infection-by-2030-with-united-kingdom-italy-and-spain-among-those-joining-the-list/ (2022) (Accessed 23 May 2022).

[3] The World Health Organization. *Guidelines for The Care and Treatment of Persons Diagnosed with Chronic Hepatitis C Virus Infection*. http://www.ncbi.nlm.nih. gov/books/NBK531733/ (2018) (Accessed 23 May 2022). 30307724

[4] Boeke CE (2020). Initial success from a public health approach to hepatitis C testing, treatment and cure in seven countries: The road to elimination. BMJ Glob. Health..

[5] The WHO Reginal Office for the Western Pacific. *Expert Consultation on Viral Hepatitis Elimination in the Western Pacific Region*. http://apps.who.int/iris/bitstream/handle/ 10665/205846/B5051.pdf (2021) (Accessed 3 June 2022).

[6] Our E, Trickey A, Shirali R, Kanters S, Easterbrook P (2021). Decentralisation, integration, and task-shifting in hepatitis C virus infection testing and treatment: A global systematic review and meta-analysis. Lancet Glob. Health..

[7] Castro R (2020). Effectiveness of implementing a decentralized delivery of hepatitis C virus treatment with direct-acting antivirals: A systematic review with meta-analysis. PLoS ONE.

[8] European Association for the Study of the Liver (2020). EASL recommendations on treatment of hepatitis C: Final update of the series. J. Hepatol..

[9] Markby J (2021). Assessing the impact of simplified HCV care on linkage to care amongst high-risk patients at primary healthcare clinics in Malaysia: A prospective observational study. BMJ Open.

[10] Chan HK, Hassali MA, Mohammed NS, Azlan A, Hassan MRA (2022). Barriers to scaling up hepatitis C treatment in Malaysia: A qualitative study with key stakeholders. BMC Public Health.

[11] Ministry of Health Malaysia. *Management of Chronic Hepatitis C in Adults*. https://www.moh.gov.my/moh/resources/Penerbitan/CPG/Gastroenterology/QR_Management_of_Chronic_Hepatitis_C_in_Adults.pdf (2019) (Accessed 21 Feb 2022).

[12] Cheng CH (2020). Subgroup analysis of the predictive ability of aspartate aminotransferase to platelet ratio index (APRI) and fibrosis-4 (FIB-4) for assessing hepatic fibrosis among patients with chronic hepatitis C. J. Microbiol. Immunol. Infect..

[13] Suan MAM, Chan HK, Sem XH, Shilton S, Hassan MRA (2021). Comparing various cut-offs of aspartate aminotransferase-to-platelet ratio index (APRI) in liver cirrhosis diagnosis among hepatitis C patients in Malaysia. Med. J. Malaysia..

[14] Rungta S, Kumari S, Deep A, Verma K, Swaroop S (2021). APRI and FIB-4 performance to assess liver fibrosis against predefined Fibroscan values in chronic hepatitis C virus infection. J. Fam. Med. Prim. Care..

[15] Papadopoulos N (2019). Liver fibrosis staging with combination of APRI and FIB-4 scoring systems in chronic hepatitis C as an alternative to transient elastography. Ann. Gastroenterol..

[16] Wai CT (2003). A simple noninvasive index can predict both significant fibrosis and cirrhosis in patients with chronic hepatitis C. Hepatol. Baltim. Md..

[17] Vallet-Pichard A (2007). FIB-4: An inexpensive and accurate marker of fibrosis in HCV infection, comparison with liver biopsy and fibrotest. Hepatology.

[18] Castera L, Forns X, Alberti A (2008). Non-invasive evaluation of liver fibrosis using transient elastography. J. Hepatol..

[19] de Lédinghen V, Vergniol J (2008). Transient elastography (FibroScan). Gastroenterol. Clin. Biol..

[20] R Core Team. *R: A Language and Environment for Statistical Computing*. https://www.R-project.org/ (2017) (Accessed 8 Feb 2022).

[21] Altman DG, Bland JM (1994). Diagnostic tests 3: Receiver operating characteristic plots. BMJ.

[22] Lin ZH (2011). Performance of the aspartate aminotransferase-to-platelet ratio index for the staging of hepatitis C-related fibrosis: An updated meta-analysis. Hepatol. Baltim. Md..

[23] Joo SK (2015). Prospective comparison of noninvasive fibrosis assessment to predict advanced fibrosis or cirrhosis in Asian patients with hepatitis C. J. Clin. Gastroenterol..

[24] Holmberg SD (2013). Noninvasive serum fibrosis markers for screening and staging chronic hepatitis C virus patients in a large US cohort. Clin. Infect. Dis..

[25] Johnston DE (1999). Special considerations in interpreting liver function tests. Am. Fam. Physician..

[26] Yin LK, Tong KS (2009). Elevated ALT and AST in an asymptomatic person: What the primary care doctor should do?. Malays Fam. Physician..

[27] Hayashi H, Beppu T, Shirabe K, Maehara Y, Baba H (2014). Management of thrombocytopenia due to liver cirrhosis: A review. World J. Gastroenterol..

[28] Friedrich-Rust M (2008). Performance of transient elastography for the staging of liver fibrosis: A meta-analysis. Gastroenterology.

[29] Su S (2018). HBV, HCV, and HBV/HCV co-infection among HIV-positive patients in Hunan province, China: Regimen selection, hepatotoxicity, and antiretroviral therapy outcome. J. Med. Virol..

[30] Pretorius E (2021). Platelets in HIV: A guardian of host defence or transient reservoir of the virus?. Front. Immunol..

